# Laboratory Findings in Children with Excess Body Weight in Romania

**DOI:** 10.3390/medicina59020319

**Published:** 2023-02-09

**Authors:** Bogdan Mihai Pascu, Victor Daniel Miron, Emanuela Rachel Matei, Mihai Craiu

**Affiliations:** 1 Faculty of Medicine, Carol Davila University of Medicine and Pharmacy, 050474 Bucharest, Romania; 2 National Institute for Mother and Child Health “Alessandrescu-Rusescu”, 020395 Bucharest, Romania

**Keywords:** children, excess body weight, laboratory findings, obesity, overweight

## Abstract

*Background and Objectives:* Childhood obesity has been increasing at a worrisome pace and emerging as a non-infectious pandemic in the pediatric population in recent years. Raising awareness on this problem is of utmost importance, in order to take action to control body weight from an early age. *Materials and Methods:* We performed a retrospective study among overweight or obese children evaluated on an outpatient basis in the Department of Pediatric Endocrinology of a tertiary care hospital in Bucharest Romania in 2021 in order to identify laboratory changes occurring according to age and sex. *Results:* A total of 268 children were included in the analysis, with a median age of 10.9 years (IQR: 8.3, 13.3 years); 61.8% were obese and 38.2% overweight. We identified a subclinical pro-inflammatory status characterized by increased neutrophil count (12.7%) and increased C-reactive protein (16.4%). Biochemically, we identified the highest increases for uric acid (35.4%). More than half of the children included in the study had dyslipidemia-specific changes: high low-density lipoprotein cholesterol (LDL) (50.0%), low high-density lipoprotein cholesterol (HDL) (58.9%) and increased triglyceride levels (12.7%), especially children with a body mass-index (BMI) percentile above 95%. Increased thyroid stimulating hormone (TSH) was identified in 20.3% and low thyroxine (T4) level in 13.4%, especially in females. *Conclusions:* Early measures to control excess body weight are needed since preventing obesity is easier than treating it. However, this is often difficult to do in our country because parents frequently do not recognize the problem until it is advanced. Furthermore, doctors are not always adequately prepared and sometimes they do not have the support of the health systems to provide children in need with the adequate care. Educational strategies and awareness of issue should be revisited in current post-pandemic context that facilitates increase of obesity prevalence in children. Increase of efficient communication could be achieved by pointing to these objective findings.

## 1. Introduction

Childhood obesity may be considered one of the silent pandemics of the 21st century. The prevalence of childhood excess body weight is increasing year by year and has a global geographical distribution, being found in both high- and low-income countries [1]. While underweight rates among children remain constant, overweight and obesity rates are increasing at the expense of normal weight [1].

The COVID-19 pandemic played a major role in worsening the excess body weight status of children. Numerous reports show the negative impact that the pandemic has had indirectly in increasing the number of obese children [2,3,4]. Currently, in the USA, the prevalence of childhood obesity is reported to be 19.7% [5], and in the European Region one in three children are overweight or obese [6]. The situation is even more worrisome as the percentage of children under 5 with obesity is on an ascending trend, reaching 12.7% in the USA, according to the Centre for Disease Control and Prevention [5]. Globalisation, urbanisation, changing lifestyles with lack of physical activity, increasing screen time (TV, phone, videogames), and consumption of high-calorie and ultra-processed foods are among the key determinants of obesity [7,8]. However, the problem of excess body weight is multifactorial [7,9]. The establishment of a metabolic imbalance leads to a vicious circle that becomes difficult to control and inevitably leads to the continuous accumulation of extra weight, which in turn triggers new imbalances.

In Romania, the situation is as worrisome as it is globally. In 2018, the prevalence of pediatric obesity was 18.5% [10] an alarming increase from 2010 when it was reported at around 10% [11]. Moreover, in our country the phrase “chubby and beautiful” is still used with great enthusiasm by parents and grandparents and many of them do not recognize that being overweight or obese represents a problem and, consequently, are not willing to actively implement weight loss activities [10].

Under the circumstances, it is imperative to raise awareness among parents, and the population in general, of the implications of excess body weight in children. Obesity is not just a problem of physical appearance, its repercussions on health are much more profound, as it induces imbalances that persist and will worsen during adulthood [12]. In addition, doctors, especially pediatricians and general practitioners, must be trained to identify causes and risk factors, to actively communicate with parents and children, and to develop a personalized action plan within a multidisciplinary team. To this end, the aim of the current study was to identify and highlight changes in laboratory parameters in children with excess body weight according to age and sex in our country, hoping to bring new communication objective arguments.

## 2. Methods

We conducted a retrospective study among children evaluated for weight-related problems in the Department of Pediatric Endocrinology (DPE) of the National Institute for Mother and Child Health “Alessandrescu-Rusescu” (NIMCH) during the period 1 January–31 December 2021.

NIMCH is a tertiary pediatric hospital in Bucharest (the capital of Romania). It serves the Northern region of the city and the entire adjacent Ilfov-Pipera urban area. In the DPE pediatric outpatients are evaluated, and in 2021 about 4000 children underwent assessment here, of whom about 1400 with weight problems.

Inclusion criteria for the study were (i) child aged 2–18 years presenting to the DPE between 1 January and 31 December 2021; (ii) being overweight (body mass index—BMI—percentile above 85% but below 95%) or obese (BMI percentile above 95%); (iii) assessed with laboratory investigations in NIMCH at the time of presentation; and (iv) had no signs or symptoms of acute illness, or chronic diseases (metabolic, cardiac, pulmonary, renal, hepatic, genetic diseases, etc.), hormonal diseases, short stature, medication, or genetic obesity.

Children under 2 years of age, those with a BMI percentile below 85%, those who had not been investigated with laboratory tests at the time of the initial assessment, and those whose personal records showed signs and symptoms of acute illness or other documented chronic conditions (see above) that could have influenced the current analysis were excluded.

For each patient, two of the authors collected from the hospital’s information system age, sex, weight, height, and laboratory investigation data (blood count, biochemistry, inflammation markers, thyroid function). The Centre for Disease Control and Prevention [13] calculator was used to calculate percentiles for BMI. Interpretation of laboratory investigations was done by comparing the patient’s results with the reference ranges (upper or lower limit of normal) for age and sex used by the hospital’s laboratory. Normal laboratory ranges are detailed in Table S1. Clinical examination and measurement of weight and height was done by the same pediatric endocrinologist and the same measuring device (electronic scale with tally meter, ADE, Hamburg, Germany) was used for all patients included in the study. The children included in the study were divided into three age groups: pre-schoolers, 2–5 years (24–60 months); school children, 5–14 years (61–168 months); and adolescents, 14–18 years (169–216 months).

Statistical analysis was performed using IBM SPSS Statistics for Windows, version 25 (IBM Corp., Armonk, NY, USA). After applying the Kolmogorov–Smirnov test, as all our continuous variables did not have normal distribution, we reported median and IQR (25th–75th percentiles) and Mann–Whitney U test results for comparative analysis. We registered the qualitative dichotomous data as frequencies and percentages and for comparative analysis we reported the results of the chi-square test with odds ratios and 95% confidence interval (CI). Statistical significance was set at *p* < 0.05.

## 3. Results

### 3.1. General Data Analysis

**Figure 1:**
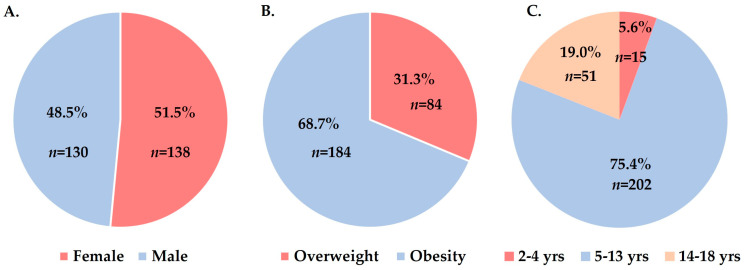
Distribution of children by sex (**A**), type of excess body weight (**B**), and age group (**C**).

A total of 268 children simultaneously met all inclusion criteria and were included in the study: 51.5% (*n* = 138) were female, and 68.7% (*n* = 184) were obese. The median age was 10.9 years (IQR: 8.3, 13.3 years), with the majority of children being in the 5–14 age group (75.4%, *n* = 202) (Figure 1).

**Figure 2:**
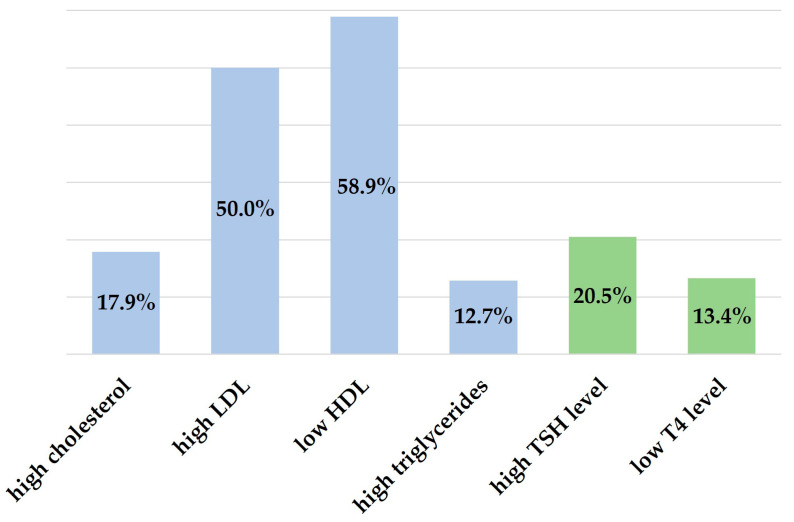
Lipid profile and hormonal changes in the study group.

We identified a number of changes in laboratory parameters in pediatric patients with excess body weight that we summarized in Table 1. Increased neutrophils were present in 12.7% of children, 15.3% had anemia, and 9.0% had increased platelets. Biochemically we identified increases in alkaline phosphatase (ALP) and uric acid in 13.4% and 35.4% of children, respectively. Lipid profile showed dyslipidemia-specific changes in a high number of children (Figure 2). Of these, 9 (3.4%) children showed consistent changes throughout all lipid profile markers (high cholesterol, high low-density lipoprotein (LDL), low high-density lipoprotein (HDL), high triglycerides), of which 5 were female, 7 were school children (5–13 years), and 2 adolescents (14–18 years), and 6 were obese with BMI percentiles above 95%. A percentage of 20.5% of the children had changes in TSH serum levels and 13.4% had low thyroxine (T4) values (Figure 2). Inflammatory syndrome was detected in an important number of children, 44 (16.4%) had increased C-reactive protein (CRP) and 61 (22.8%) had increased erythrocyte sedimentation rate (ESR).

### 3.2. Analysis of Data According to Sex and Age Group

Overall, boys had a 2.1-fold higher risk of being obese (76.9% vs. 60.9%, *p* = 0.005, χ^2^ = 8.0, OR = 2.1, 95% CI: 1.3–3.6). We found no differences between median ages by sex (11.0 years; IQR: 8.9–13.0 years) for boys and 10.1 years (IQR: 7.8–13.6 years) for girls, *p* = 0.508). In age group analysis, overweight girls predominated (22.5% vs. 15.4%) among adolescents (14–18 years), Table 2. We identified a high proportion of obesity in young children under 5 years of age (86.7%), Table 2.

Dynamics of laboratory findings by sex and age groups are shown in Table 3. Girls had a 3.9-fold increased risk of low T4 values (*p* < 0.001, χ^2^ = 11.5, OR = 3.9, 95% CI: 1.7–8.9) and a 2.1-fold increased risk of high ESR values (*p* = 0.012, χ^2^ = 6.3, OR = 2.1, 95% CI: 1.2–8.9), regardless of age (Table 3). Adolescents had a 5.1-fold higher risk of hyperuricemia than the other age groups, regardless of sex (*p* < 0.001, χ^2^ = 26.8, OR = 5.1, 95% CI: 2.7–9.8).

### 3.3. Data Analysis: Overweight vs. Obesity

The analysis of laboratory parameters according to the type of excess body weight is shown in Table 4. We identified that, when compared to overweight, obesity is associated with a 4.4-fold higher risk of uric acid increases (*p* < 0.001, χ^2^ = 21.3, OR = 4.4, 95% CI: 2.3–8.5), a 2.5-fold higher risk of ALP elevations (*p* = 0.041, χ^2^ = 4.5, OR = 2.5, 95% CI: 1.01–6.3), a 1.9-fold higher risk of having high LDL levels (*p* = 0.017, χ^2^ = 5.6, OR = 1.9, 95% CI: 1.1–3.2), and a 2.1-fold higher risk of having low HDL levels (*p* = 0.004, χ^2^ = 7.9, OR = 2.1, 95% CI: 1.3–3.6). Children with obesity also had a 2.8-fold higher risk of having inflammatory syndrome with increased CRP levels (*p* = 0.015, χ^2^ = 5.8, OR = 2.8, 95% CI: 1.2–6.5).

## 4. Discussion

The implications of obesity for children are extensive, from short- and long-term health problems to social and psychological problems and low self-esteem. In addition, up to 80% of obese children remain overweight into adulthood [12]. Measures to control body weight in children need to be taken as soon as possible; however, this can only be done after parents and doctors become truly aware of the impact of obesity on children. Our study aimed to provide a useful tool in medical practice. We tried to highlight changes in laboratory parameters that occur among children with overweight or obesity, to be used as a tool to validate the information provided in discussions with parents.

Children who were assessed in the PED for problems related to excess body weight were included in the analysis. To maximise the accuracy of the data, we excluded possible factors such as acute or chronic diseases that could have altered the parameters being tracked.

Over-accumulation of adipose tissue leads to mild systemic and chronic inflammation [14]. Among white blood cells (WBC), recent studies have shown that neutrophils are the first immune cells to reach the adipose tissue [15], implying an increase in circulating neutrophil levels as well, which is directly proportional to the severity of obesity [16,17,18]. Dixon et al. showed as early as 2006 that weight loss in obese patients also led to significant decreases in WBCs and neutrophils, while increases in BMI led to increases in neutrophils [19]. Overall, the physiological roles of neutrophils in infection and inflammation are well known. In obese adult patients, increased neutrophil values have been associated with carotid atherosclerosis, impaired glucose tolerance and microvascular and macrovascular complications of type II diabetes [15,20]. We identified a high percentage of children with excess body weight and neutrophilia (12.7%), and a higher median value compared to other reports [21].

Increased levels of interleukin-6 (IL-6) from adipose tissue are also involved in promoting thrombopoietin synthesis, thus stimulating megakaryocytopoiesis. Samocha-Bonet et al. showed that platelet counts are higher in obese adult patients and neutrophil counts correlate directly with BMI [22]. However, there is no evidence showing an increase in thrombocyte activation in obese patients [22,23]. Our data on platelet values are similar to another study published in Romania in pediatric patients with obesity [21], but higher than a report from South Korea [24].

Obesity-driven chronic inflammation can reduce iron bioavailability, which can in turn translate into iron deficiency anemia in obese patients [25]. In our study we identified that 15.3% of overweight and obese children presented anemia, without a direct correlation with age or sex. Another study showed that the risk of iron deficiency anemia for obese children and adolescents is relatively equal to that of the normal-weight children and adolescents [24]. Overall, iron deficiency is common in overweight and obese children [26].

The best and easiest indicator in clinical practice for quantifying inflammatory status is CRP. In 2018, de Dios et al. showed that adipose tissue was a major extra-hepatic source for CRP synthesis [27]. Moreover, several studies have shown a positive association between obesity, type 1 diabetes, and increased serum CRP levels [28,29,30]. In contrast, Cayres et al. showed that adolescents with regular physical activity had lower serum CRP values compared to sedentary adolescents [31]. In our study, 16.4% of children had increased CRP values, and these were significantly associated with obesity (percentiles for BMI above 95%).

Increased insulin resistance and the development of type 2 diabetes is an important consequence of pediatric obesity, which routinely occurs in adulthood as a result of ineffective weight control [32]. Metabolic disorders are accompanied by adipocyte accumulation in other parenchymal organs, of which the liver is the most commonly affected. Unfortunately, non-alcoholic fatty liver disease (NAFLD) is increasingly reported in children with obesity, its prevalence being almost 10 times higher than among children with normal BMI [33]. Furthermore, a complex interaction between inflammation and NAFLD has been shown among children with obesity, for which inflammatory cytokines, such as IL-1β, IL-6 and IL-17, have been recently described as markers for NAFLD risk [34]. This leads to mild but persistent hepatic cytolysis [35]. Of the 268 children included in the study, 8.6% had increased AST and 7.5% increased ALT values. Children with obesity should have regular follow-up of transaminases and ultrasound liver evaluation [36].

We identified a high percentage of children with hyperuricemia, especially among adolescents. This has also been shown in other studies. For example, Rospleszcz et al. showed that patients with abdominal obesity had an increased risk for hyperuricemia [37], and Li et al. found that obesity and insulin resistance were independent factors for hyperuricemia [38].

A significant finding of our study is the high rate of dyslipidemia among overweight children. This change plays an important role in the development of coronary heart disease, a major cause of mortality and morbidity in patients with excess body weight. Therefore, detection and treatment of dyslipidemia should start in childhood so that lipid levels are maintained within normal limits. Kwiterovich P. defines childhood obesity-associated dyslipidemia as increased triglycerides or high LDL or low HDL [39]. We identified significant changes in LDL and HDL levels in more than 50% of the overweight or obese children in our routine clinical practice, and increased triglycerides in 12.7% of them. Numerous studies from other countries have reported similar data [40,41,42]. Prior to drug treatment to normalize lipid levels, lifestyle modification through balanced diet and regular physical activity are key factors in managing these metabolic imbalances.

Thyroid hormones are involved in the regulation of energy homeostasis, lipid oxidation and lipid and glucose metabolism. Therefore, high TSH levels can be considered an indicator of altered energy balance in obesity [43]. We identified in our study that 20.5% of children had increased serum TSH levels, and 13.4% had low serum T4 levels. Although the pathophysiology of this relationship is not fully understood, the decrease in TSH levels following weight loss confirms the reversible nature of this change [44]. The documented prevalence of increased TSH in children with obesity is between 1% and 21% and has been associated with higher triglycerides, LDL, and total cholesterol [44]. Monitoring of thyroid function is absolutely necessary in all children with excess body weight regardless of age and sex.

Monitoring of excess body weight in children is very important, as obesity is recognised as a risk factor, including for acute illnesses. Thus, in recent years we have seen that obesity has predisposed to severe COVID-19 [45], but there is data also showing that excess body weight children develop more severe forms of influenza [46] and are more susceptible to urinary tract infections [47]. Therefore, interventions need to occur early, a healthy lifestyle (proper nutrition, regular physical activity, reduced screen time, etc.) should be instituted from an early age. In addition, overweight children should benefit from medical and psychological counselling, and active prophylactic methods, such as vaccination to prevent acute diseases that can prove to be severe in this group of patients [48,49].

Our study has a number of limitations, represented by the retrospective nature of the data and the lack of follow-up of the children included in the study to document the dynamic patterns of laboratory parameters in relation to the evolution of the weight trend. However, the large number of children and the exclusion of all major factors that could have altered the parameters studied make our study provide a comprehensive overview of the impact of obesity on children in Romania. Our data shows that for our culture and geographic region, the phrase ”chubby and beautiful” is neither appropriate nor true. The excess body weight Romanian children show a series of metabolic and inflammatory changes from an early age. Over time, their persistence may worsen and in turn lead to the appearance of other imbalances that will be difficult to correct in adulthood. Thus, our study is a starting point and a warning signal about the ”unseen implications” of excess body weight in Romanian children. Our observations may form the basis for future studies on larger groups of patients and with long-term follow-up.

## 5. Conclusions

We identified a number of important changes in laboratory parameters in children with excess body weight. We have shown that dyslipidemia, impaired thyroid function, and a sub-clinical inflammatory status are present in overweight and obese children and occur from an early age. Early measures to control overweight are needed because preventing obesity is easier than treating it. However, this is often difficult to do in our country because parents frequently do not recognize the problem until it is advanced. Furthermore, doctors are not always adequately prepared and some-times they do not have the support of the health systems to provide children in need with the ad-equate care. Educational strategies and awareness of issue should be revisited in current post-pandemic context that facilitates increase of obesity prevalence in children. Increase of efficient communication could be achieved by pointing to these objective findings.

## Figures and Tables

**Table 1:** Laboratory findings in the study group.

Lab Analysis		Results	Unit Lab
WBC	increased, *n* (%)	2 (0.7)	
	value, median (IQR)	8.82 (6.08, 10.0)	×10^3^/μL
Neutrophils	increased, *n* (%)	34 (12.7)	
	value, median (IQR)	5.08 (3.53, 6.01)	×10^3^/μL
Hemoglobin	decreased, *n* (%)	41 (15.3)	
	value, median (IQR)	12.8 (12.4, 13.0)	g/mL
Platelets	increased, *n* (%)	24 (9.0)	
	value, median (IQR)	347 (323, 389)	×10^3^ μL
Blood glucose	increased, *n* (%)	9 (3.4)	
	value, median (IQR)	85 (79, 89)	mg/dL
AST	increased, *n* (%)	23 (8.6)	
	value, median (IQR)	28 (23, 32)	U/L
ALT	increased, *n* (%)	20 (7.5)	
	value, median (IQR)	27 (21, 34)	U/L
ALP	increased, *n* (%)	36 (13.4)	
	value, median (IQR)	303 (235, 352)	mg/dL
Urea	increased, *n* (%)	27 (10.0)	
	value, median (IQR)	23 (17, 31)	mg/dL
Creatinine	increased, *n* (%)	0 (0.0)	
	value, median (IQR)	0.5 (0.5, 0.6)	mg/dL
Uric acid	increased, *n* (%)	95 (35.4)	
	value, median (IQR)	4.7 (4.1, 5.3)	mg/dL
Cholesterol	increased, *n* (%)	48 (17.9)	
	value, median (IQR)	141 (126, 176)	mg/dL
LDL	increased, *n* (%)	134 (50.0)	
	value, median (IQR)	90 (69, 112)	mg/dL
HDL	decreased, *n* (%)	158 (58.9)	
	value, median (IQR)	47 (41, 54)	mg/dL
Triglycerides	increased, *n* (%)	34 (12.7)	
	value, median (IQR)	92 (64, 129)	mg/dL
TSH	increased, *n* (%)	55 (20.5)	
	value, median (IQR)	2.74 (2.34, 5.54)	μIU/dL
T4	decreased, *n* (%)	36 (13.4)	
	value, median (IQR)	1.06 (0.84, 1.13)	ng/dL
CRP	increased, *n* (%)	44 (16.4)	
	value, median (IQR)	0.22 (0.06, 0.33)	mg/dL
ESR	increased, *n* (%)	61 (22.8)	
	value, median (IQR)	10 (8, 14)	mm/h

WBC—white blood cells; AST—aspartate aminotransferase; ALT—alanine aminotransferase; ALP—alkaline phosphatase; LDL—low-density lipoprotein; HDL—high-density lipoprotein; TSH—thyroid stimulating hormone; T4—thyroxine; CRP—C-reactive protein; ESR—erythrocyte sedimentation rate.

**Table 2:** Distribution of children by gender and type of excess body weight according to age group.

Age Group	Sex		*p*-Value	Type of Excess Body Weight		*p*-Value
	Female, *N* = 138, n (%)	Male, *N* = 130, *n* (%)		Overweight *N* = 84, *n* (%)	Obesity, *N* = 202, *n* (%)	
2–5 years	10 (7.2)	5 (3.8)	0.226	2 (2.4)	13 (6.4)	0.245
5–14 years	97 (70.3)	105 (80.8)	0.046 *	62 (73.8)	140 (69.3)	0.446
14–18 years	31 (22.5)	20 (15.4)	0.139	20 (23.8)	31 (15.3)	0.088

* χ^2^ = 3.96, OR = 1.8, 95% CI = 1.005-3.1.

**Table 3:** Laboratory findings according to sex and age group.

Lab Analysis	Sex		*p*-Value	Age Group			*p*-Value
	Female, *N* = 138, *n* (%)	Male, *N* = 130, *n* (%)		2–5 yrs *N* = 15, *n* (%)	5–14 yrs, *N* = 202, *n* (%)	14–18 yrs, *N* = 51, *n* (%)	
WBC increase	1 (0.7)	1 (0.7)	NA	1 (6.7)	1 (0.5)	0 (0.0)	NA
Neutrophils increase	21 (15.2)	13 (10.0)	0.198	4 (26.7)	19 (9.4)	11 (21.6)	0.075
Anemia	22 (15.9)	19 (14.6)	0.764	0 (0.0)	30 (14.9)	11 (21.6)	0.117
Platelets increase	13 (9.4)	11 (8.5)	0.777	3 (20.0)	17 (8.4)	4 (7.8)	0.302
High blood glucose	3 (2.2)	6 (4.6)	0.286	0 (0.0)	8 (4.0)	1 (2.0)	0.588
AST increase	9 (6.5)	14 (10.8)	0.214	4 (26.7)	15 (7.4)	4 (7.8)	0.203
ALT increase	7 (5.1)	13 (10.0)	0.125	2 (13.3)	10 (5.0)	8 (15.7)	0.689
ALP increase	16 (12.0)	20 (15.4)	0.362	0 (0.0)	30 (14.9)	6 (11.8)	0.765
Urea increase	10 (7.2)	17 (13.1)	0.113	2 (13.3)	21 (10.4)	4 (7.8)	0.759
Uric acid increase	42 (30.4)	53 (40.8)	0.077	1 (6.7)	60 (29.7)	34 (66.7) ^‡^	<0.001
High cholesterol	27 (19.6)	21 (16.2)	0.466	1 (6.7)	36 (17.8)	11 (21.6)	0.526
High LDL	70 (50.7)	64 (49.2)	0.806	5 (33.3)	106 (52.5)	23 (45.1)	0.315
Low HDL	74 (53.6)	84 (64.6)	0.067	9 (60.0)	115 (56.9)	34 (66.7)	0.549
High TG	18 (13.0)	16 (12.3)	0.862	1 (6.7)	26 (12.9)	7 (13.7)	0.926
TSH increase	28 (20.3)	27 (20.8)	0.920	1 (6.7)	41 (20.3)	13 (25.5)	0.378
Low T4 level	28 (20.3) ^‡^	8 (6.2)	<0.001	0 (0.0)	26 (12.9)	10 (19.6)	0.535
CRP increase	25 (18.1)	19 (14.6)	0.438	3 (20.0)	33 (29.2)	8 (15.7)	0.877
ESR increase	40 (29.0) ^‡^	21 (16.2)	0.012	0 (0.0)	48 (23.8)	13 (25.5)	0.541

WBC—white blood cells; AST—aspartate aminotransferase; ALT—alanine aminotransferase; ALP—alkaline phosphatase; LDL—low-density lipoprotein; HDL—high-density lipoprotein; TSH—thyroid stimulating hormone; T4—thyroxine; CRP—C-reactive protein; ESR—erythrocyte sedimentation rate; ^‡^ group with statistical significance.

**Table 4:** Laboratory findings according to type of excess body weight.

Lab Analysis	Overweight, *N* = 84, *n* (%)	Obesity, *N* = 184, *n* (%)	*p*-Value
WBC increase	0 (0.0)	0.0 (1.1)	N/A
Neutrophils increase	8 (9.5)	26 (14.1)	0.450
Anemia	10 (11.9)	31 (16.8)	0.297
Platelets increase	5 (6.0)	19 (10.3)	0.245
High blood glucose	2 (2.4)	7 (3.8)	0.639
AST increase	6 (7.1)	17 (9.2)	0.571
ALT increase	4 (4.8)	16 (8.7)	0.256
ALP increase	6 (7.1)	30 (16.3) ^‡^	0.041
Urea increase	10 (11.9)	17 (9.2)	0.502
Uric acid increase	13 (15.5)	82 (44.6) ^‡^	<0.001
High cholesterol	13 (15.5)	35 (19.0)	0.483
High LDL	33 (39.3)	101 (54.9) ^‡^	0.017
Low HDL	39 (46.2)	119 (64.7) ^‡^	0.004
High TG	7 (8.3)	27 (14.7)	0.148
TSH increase	15 (17.9)	40 (21.7)	0.466
Low T4 level	10 (11.9)	26 (14.1)	0.617
CRP increase	7 (8.3)	37 (20.1) ^‡^	0.015
ESR increase	19 (22.6)	42 (22.8)	0.935

WBC—white blood cells; AST—aspartate aminotransferase; ALT—alanine aminotransferase; ALP—alkaline phosphatase; LDL—low-density lipoprotein; HDL—high-density lipoprotein; TSH—thyroid stimulating hormone; T4—thyroxine; CRP—C-reactive protein; ESR—erythrocyte sedimentation rate; ^‡^ group with statistical significance.

## Data Availability

The datasets generated and analysed during the current study are available from the corresponding author upon reasonable request.

## Supplementary Materials

The following are available online at https://www.mdpi.com/article/10.3390/medicina59020319/s1, Table S1: Normal laboratory range.
