# Supplementary material for: Laboratory Findings in Children with Excess Body Weight in Romania
Source: Medicina (Kaunas). 2023 Feb 9;59(2):319. doi: 10.3390/medicina59020319 (PMC9964917; doi:10.3390/medicina59020319)
Supplement: Supplementary file 1 [file medicina-59-00319-s001.zip › medicina-2147740-supplementary.pdf]

Supplementary material

Table S1. Normal laboratory range

Lab parameters	Unit	Age groups		
		**2-5 years**	**5-14 years**	**14-18 years**
WBC	cells/μL	$5 - 14 \times 10^3$	$5 - 12 \times 10^3$	$5 - 12 \times 10^3$
Neutrophils	cells/μL	$1.5 - 8.5 \times 10^3$	$1.5 - 8.0 \times 10^3$	$1.5 - 8.0 \times 10^3$
Hemoglobin	g/dL	11 - 14	12 - 14	13 - 15
Platelets	cells/μL	$150 - 450 \times 10^3$	$150 - 450 \times 10^3$	$150 - 450 \times 10^3$
Blood glucose	mg/dL	70 - 100	70 - 100	70 - 100
AST	U/L	10 - 50	10 - 37	10 - 37
ALT	U/L	10 - 60	10 - 60	10 - 60
ALP	U/L	50 - 350	50 - 500	50 - 500
Urea	mg/dL	15 - 35	15 - 35	15 - 35
Creatinine	mg/dL	0.2 - 1	0.2 - 1	0.4 - 1.4
Uric acid	mg/dL	2.0 - 5.0	2.0 - 5.0	2.0 - 5.0
Cholesterol	mg/dL	95 - 190	110 - 190	140 - 200
LDL	mg/dL	40 - 100	40 - 100	40 - 100
HDL	mg/dL	50 - 90	50 - 90	50 - 90
Triglyceride	mg/dL	30 - 160	30 - 160	30 - 160
TSH	μIU/mL	0.7 - 5.97	0.5 - 4.3	0.5 - 4.3
T4	ng/dL	0.85 - 1.77	0.80 - 1.75	0.80 - 1.75
CRP	mg/dL	0.0 - 0.5	0.0 - 0.5	0.0 - 0.5
ESR	mm/h	0 - 15	0 - 15	0 - 15

WBC - white blood cells; AST - aspartate aminotransferase; ALT - alanine aminotransferase; ALP - alkaline phosphatase; LDL - low-density lipoprotein; HDL - high-density lipoprotein; TSH - thyroid stimulating hormone; T4 – thyroxine; CRP - C-reactive protein; ESR - erythrocyte sedimentation rate.
